# MusicCohort: Pilot feasibility of a protocol to assess students’ physical and mental health in a Canadian post-secondary school of music

**DOI:** 10.1186/s13104-021-05829-9

**Published:** 2021-12-04

**Authors:** Julius Bruder, Nikolaus Ballenberger, Bethany Villas, Charlotte Haugan, Kimiko McKenzie, Zalak Patel, Christoff Zalpour, Amynah Mevawala, Melisa Handl, Christine Guptill

**Affiliations:** 1 Department of Occupational Therapy, Faculty of Rehabilitation Medicine, University of Alberta, Edmonton, Canada; 2 Department of Economics and Social Sciences, Hochschule Osnabrück, Albrechtstr. 30, 49076 Osnabrück, Germany; 3 Faculty of Law, University of Ottawa, Ottawa, Canada; 4 School of Rehabilitation Sciences, Faculty of Health Sciences, University of Ottawa, 3071 Guindon Hall, 451 Smyth Road, Ottawa, ON K1H 8M5 Canada

**Keywords:** Musicians, Music students, Assessment protocol, Feasibility, Mental health, Physical health

## Abstract

**Objective:**

Music-related physical and mental health conditions are common among post-secondary music students, with many studies reporting a prevalence greater than 70%. However, there is currently no consensus on appropriate, validated assessments for this population. The aim of this pilot study was to test the feasibility of an assessment protocol developed for a German longitudinal study with Canadian post-secondary music students, and to compare the health of music students to non-music students. Using a cross-sectional design, first-semester music and non-music control students were recruited at two campuses at the same university. Both groups completed questionnaires and physical testing, including range of motion, core strength, and pressure pain threshold. Nineteen music students and 50 non-music student controls participated in this study.

**Results:**

The German protocol is feasible in a Canadian post-secondary setting. Canadian music students demonstrated similar health outcomes to those in the parent study. All participants demonstrated poorer mental and physical quality of life than the Canadian norms, though this was not statistically significant. The results of this study should be confirmed in a larger study. Future studies with larger sample sizes can provide further insight into the health of Canadian music students, providing a basis for prevention and intervention.

**Supplementary Information:**

The online version contains supplementary material available at 10.1186/s13104-021-05829-9.

## Introduction

Post-secondary music programs place significant physical and psychological demands on students. Studies report the prevalence of Playing Related Musculoskeletal Disorders (PRMD) [1] as greater than 70% in post-secondary music students [2–7]. High prevalence of stress, depression, and anxiety are also reported [2, 5, 8–10]. Research in this field is quite heterogeneous, with definitions of music-related health conditions varying significantly. More consistency is needed, including the development and use of valid, occupation-specific assessment and measurement tools [11]. To this end, a new protocol to assess music students' health throughout their degree was developed in Germany [12]. The protocol was designed to use current, validated tools, while minimizing the time required for administration for both the tester and the subject. Both physical and mental health measures were included, since the literature indicates that musicians’ mental and physical health are interrelated [13–15].

The primary goal of this pilot study was to test the feasibility of the German protocol in a Canadian music program [16, 17]. In particular, we aimed to ensure that assessment procedures were reproduceable, that all necessary resources were available, and we aimed to reduce the protocol from its initial length of 90 min, to permit administration in a typical 1-h initial therapy appointment [16]. A secondary goal was to compare the health of Canadian music students to non-music students.

## Main text

### Methods

#### Study design

A cross-sectional study was conducted with full-time, first year undergraduate students at a university in Western Canada. Inclusion criteria were: (1) Bachelor of Music (majoring in music) (case) or majoring in any other program (control), and (2) age 16 or older. Exclusion criteria were: (1) diagnosed neurological, orthopaedic, or psychological condition, (2) infection or systemic disease, (3) regular medication for mental illness or pain, (4) varsity (inter-university) athlete, (5) for controls, enrollment in music courses where a grade is assigned for music performance.

### Recruitment

Recruitment took place from September to November 2016–2018, and March to May 2018. Data from 19 music students and 50 controls were analyzed (n = 69). No sample size calculation was conducted for this pilot feasibility study. Ethics approval was obtained from the university’s health research ethics board. Assessment instruments, required equipment, and details about recruitment and incentives are described in the Additional file 1: (S1–4).

### Statistical analysis

IBM SPSS version 25 and Microsoft Excel 2013 were used to analyze data, with alpha set at p < 0.05. Most characteristics were not normally distributed, so when comparing music students to controls, Mann–Whitney U (continuous) and Chi-square (categorical) tests were employed. Preliminary analyses showed that music and control groups were comparable in terms of age and gender, so data were not adjusted for these variables. Effect size r was calculated by dividing the z-value by square root of N [18].

## Results

### General demographics

Gender distribution was similar in both groups, with 68.40% female among music students and 62.00% in controls. Average course load in hours per week for music students and controls was 18.87 + 3.30 and 18.64 + 5.20, respectively. In addition, music students spent an average of 24.76 (SD 9.898) hours playing either their primary or secondary instrument.

Music students’ self-reported health behaviour scores were significantly lower than controls for nutrition (p = 0.022) and physical activity (p = 0.001). Participants were grouped into those who reported physical activity (PA) at/above the suggested 90 min per week [19], and those who reported lower PA. Analysis showed that musicians were significantly less likely to engage in PA (χ^2^ = 6.25, p = 0.016, OR 4.20). Both groups were comparable for the remaining characteristics (see Table 1).

**Table 1:** General demographics

	Total	Musicians	Control	Statistics
Total	69	19	50	
Female gender	44 (63.76%)	13 (68.42%)	31 (62.00%)	p = 0.781, X^2^ = 0.246
Age, years	18.00 (2.00)	18.00 (2.00)	18.00 (0.00)	p = 0.480, r = −0.085, Z = −0. 707
Height, cm	164.50 (0.12)	164.5 (12.00)	166.50 (13.00)	p = 0.961, r = −0.049, Z = −0.049
Body weight, kg	59.5 (10.50)	59.5 (10.50)	61.00 (13.75)	p = 0.829, r = −0.216, Z = −0.216
BMI	21.77 (5.30)	21.77 (3.06)	21.70 (2.85)	p = 0.565, r = −0.070, Z = −0.578
Sleep duration, hr	7.00 (0.63)	7.00 (0.63)	7.00 (1.44)	p = 0.309, r = −0.124, Z = −1.028
Course hours/wk	17.75 (5.80)	17.75 (5.80)	18.00 (4.00)	p = 0.941, r = −0.010, Z = −0.080
Physical activity	1.25 (2.80)	1.25 (2.8)	4.00 (4.00)	p = 0.001^*^, r = −0.391, Z = −3.249
Alcohol glasses/wk	0.25 (1.30)	0.25 (1.3)	0.00 (0.90)	p = 0.321, r = −0.123, Z = −1.002
Nutrition	6.00 (1.30)	6.00 (1.30)	7.00 (2.00)	p = 0.022^*^, r = −0.274, Z = −2.281
Instrument Playing hours (musicians only)	NA	24.50 (IQR 17.6)	N/A	N/A

Values represent median and interquartile range or frequency and percentage for each respective group, test statistics for Man-Whitney-U-Test, α = 0.05, Bold * indicates α < 0.05, r = effect size

### Self-reported mental health

**Fig. 1:**
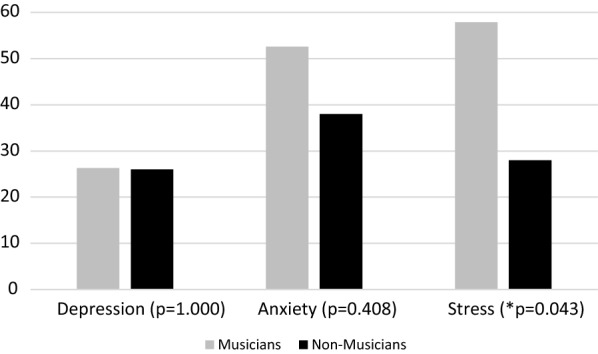
Frequency (%) of symptomatic participants by DASS21 domain

Music students had lower mental health scores in all three domains of the DASS-21 [20–22]. However, only the stress domain was statistically different (p = 0.043). Female students' scores were lower, however, this difference was not statistically significant (see Fig. 1). Over half of the tested music students (63.60%) scored above the suggested cut-off of 84 on the KMPAI-R [23–25], indicating more severe symptoms of Music Performance Anxiety, with a mean score of 94.27 (+56.64).

### Self-reported quality of life (RAND 12 [26, 27])

Differences between music students and controls were statistically significant for Body Pain (p = 0.004) and approached significance for General Health (p = 0.053) (see Additional file 1: S5). No other differences were significant.

### Pain

The 7-day point prevalence of playing-related musculoskeletal problems (PRMP) as reported on the MPIIQM [28, 29] was 31.58%, whereas lifetime prevalence was 68.42%. The most prevalent pain location was the right forearm (N = 4). Right wrist, shoulder–neck region, and left and right hand (N = 3, respectively) were the next most frequently reported. Participants reported a mean pain intensity of 4.06 on a Likert scale of 0–10. Mean pain interference was 8.53 on a scale of 0–10.

### Mobility

There were no significant ROM differences between music student participants and controls. Furthermore, participants' ROM did not differ significantly from the norms (see Additional file 1: S6). Hand span was not statistically significantly different between music students and controls; however, music students did have larger hand span for all four measurements (digit one-to-five and two-to-five on both hands). Both the Beighton score [30] and Sitting-Rising Test [31, 32] did not show significant differences between music students and controls.

### Mechanosensitivity

Music students had lower mechanical pressure pain threshold for every testing point (see Additional file 1: S7). Females had consistently lower pain thresholds than males, but these were not statistically significant.

### Core strength

Musicians demonstrated lower core strength; all but the left plank were significantly different. It is noteworthy that the control group also performed below the norm [33] (see Table 2).

**Table 2:** Core endurance

Test	Total^a^	Musician^a^	Control^a^	Statistics
Right side plank	49.00 (45.00)	30.00 (37.00)	56.50 (38.64)	p = 0.008*, r = −0.315, Z = −2.614
Left side plank	49.00 (40.50)	29.00 (35.00)	53.50 (34.25)	p = 0.054, r = −0.240, Z = −1.995
Full plank	69.00 (60.00)	52.00 (67.00)	74.00 (55.75)	p = 0.046*, r = −0.232, Z = −1.928
Biering-Sorensen-test [58]	111.00 (69.00)	80.00 (69.00)	123.00 (49.50)	p = 0.009*, r = −0.313, Z = −2.599

^*^Values represent median and interquartile range, statistics for Mann–Whitney-U-test, α = 0.05, Bold * indicates α < 0.05, r = effect size

## Discussion

### Primary goal: feasibility

Acceptability, integration and expansion [17]: Despite our best recruitment efforts described in the Additional file 1: (S1–4), this study had a small number of music student participants, which reduces statistical power. There may be several explanations, including lab distance from the music building (15 min walk) and protocol length (70 min). Some standardized tests could be completed by participants prior to a visit, which would reduce the assessment time to 45 min. In Germany, all music students attend the university physiotherapy clinic at the beginning of their program. Program culture and ethics requirements in Canada meant that we were reliant on students' goodwill and gift card incentives to encourage participation. A cultural change towards health promotion in schools of music in North America has been recommended [34, 35], and is supported in Canadian curriculum guidelines [36]. Such a change could result in campus partnerships like the one in Germany, which could increase research participation.

Implementation and practicality [17]: The results show that the protocol is feasible in a Canadian setting. Assessment tools were available in English, which is the official language of communication at this university. In other institutions, assessment tools would need to be available in French. Equipment required for administration is typically available in Canadian physio- and occupational therapy programs, with a few small exceptions. Some programs may not have access to a cervical range of motion (CROM) device or an algometer, as these are not in common use in Canadian practice. Studies have shown that goniometry can estimate CROM almost as well as the device, so the CROM may not be necessary [37]. We did have to purchase straps to secure participants to our folding massage table (plinth) (see Additional file 1: S7). We found that the protocol was easily conducted in our university lab. If this assessment protocol were administered on-site, it would require extra time to transport and set up the equipment. Such on-site assessment might enhance recruitment in future studies.

Physio- and occupational therapists in Canada are trained to assess range of motion and strength, typically in the first year of training. We found that this training, plus an additional 2 h to review skills and specific tests in this protocol (e.g. planks; hand span measurement), was sufficient for both professionals and second-year occupational therapy students to conduct these assessments competently. Less time might be required for training experienced clinicians.

Adaptation [17]: Our procedures were adapted during data collection. For example, we learned during our study that the parent study had introduced general pain ratings. We therefore had missing data from earlier participants for this parameter. After the first cohort, we learned that a revised version of the Kenny Music Performance Inventory which had not been published in English was being used in the parent study. We therefore adopted the revised version. Hence, the first cohort completed an earlier version (KMPAI), for which no cut-off scores are available [38]. These data were therefore excluded from analysis.

### Secondary goal: compare music students to controls

The data show that Canadian music students have poorer mental and physical health than non-music controls. Longitudinal analysis is ongoing in Germany, and no in-depth comparative analysis between this study and the parent study has yet been conducted. However, we can report that Canadian music student participants had similar health profiles to the German participants [39]. In addition, the most common pain locations are consistent with a recent publication from the German parent study [40]. Despite our small sample size, these consistencies with the parent study lend credibility to our findings.

Music and control students in our study reported similar numbers of course hours per week. However, music students spent an average of 13.76 additional hours per week on personal practice. We speculate that this might partly explain why music students spent less time on physical activity. Additionally, our findings of worse self-reported nutrition, less sleep (Table 1), and higher stress (Fig. 1) might be contributing to poorer health among music students.

The 7-day PRMP point prevalence of 31.58% and lifetime prevalence of 68.42% in our sample, reported on the MPIIQM, were lower than in a study by Berque and colleagues [41]. Since their research was conducted with professional musicians, this may reflect differences between students and professionals which should be examined in future research.

Music students in our study had lower pressure pain threshold on all 18 testing points and a higher mean mechanosensitivity (p = 0.029). These music students also reported more stress and anxiety. A link between stress and pain has been suggested [12, 42]. This is an important new finding, and further research is needed. Furthermore, significant differences in mechanosensitivity were located in the forearm, left supraspinatus, and left trapezius, which are often active during instrumental performance [43, 44]. The most common symptom location reported on the MPIIQM was wrist extensors, likely reflecting activity in instrumental performance [43, 44].

The RAND 12 scores were not statistically different between musicians and controls, except for the Body Pain domain. General Health approached significance, and should be repeated in a study with more participants. The MCS scores of music students and controls were not significantly different; however, the difference of 5.77 points was higher than the minimal clinically significant difference [45, 46]. Compared to SF-36 Canadian normative data [47], both music students and controls had lower MCS scores than the norms. The literature suggests that MCS and PCS scores on the SF-12 v2 (questions are equivalent to RAND 12) are comparable to SF-36 scores [48–50], and thus, this comparison is likely valid. In addition, the mean performance anxiety score of our participants was well above the suggested cut-off score of 84 points [23]. The large variability in the KMPAI-R scores of participants in our study implies the need for a larger sample to verify this finding.

Participants who reported higher levels of stress and mechanosensitivity also reported higher Bodily Pain scores. Additionally, music students in our study engaged in significantly less Physical Activity (PA) and reported a lower pain threshold. This is consistent with the literature, which suggests that PA has a beneficial effect on pain threshold [51, 52], This finding must be viewed with caution because the RAND 12 is a self-report measure. Kreutz et al. have demonstrated that music students' self-report of their health is more optimistic than objective findings [53]. Clinicians must therefore be cautious when assessing musicians' health through self-report.

The research literature suggests gender differences in several of the measured outcomes in professional musicians [13, 54]. A preliminary analysis in this study showed a tendency for females to report poorer physical and mental health outcomes. However, these results were not statistically significant and need to be studied further, particularly given the limitations of questions related to gender used in the original German study and others [55].

## Conclusion

Our study has demonstrated that the protocol developed by our German colleagues is feasible in a Canadian population. We have also demonstrated differences between the health of post-secondary music and non-music students in Canada. Future studies with larger sample sizes can provide further insight into the effectiveness of this assessment protocol for Canadian music students.

## Limitations

Like much research in this field, our study used a cross-sectional design [10, 11, 13, 56]. A longitudinal component could be an important addition in future studies to better understand the development of music-related health conditions, or to disentangle the interaction between mental and physical health. Our study could also have been improved with a larger sample size. Such larger studies could, for example, address the assessment of instrument-specific movements and postures. It could also have been improved with intra- and interrater reliability testing, which could enhance the quality of data collected with such protocols in future.

## Supplementary Information

**Additional file 1.** Participant Questionnaire.

**Additional file 2: S1**. Paper-based assessments. **S2**. Physical Assessments. **S3**. Equipment required for assessment protocol. **S4**. Additional details regarding recruitment and inclusion/exclusion criteria. **S5**. RAND12 results for total sample and by cohort. **S6**. Range of motion. **S7**. Mechanosensitivity, measured by mechanical pressure pain threshold.

## Data Availability

The dataset supporting the conclusions of this article is included within the article and its additional file. Additional data contains sensitive health information and is unfortunately not available to share outside of the research team, as we do not have ethics approval to share the data.

## Abbreviations

PT: Physiotherapy
OT: Occupational therapy
MSc: Master
BSc: Bachelor
PhD: Doctor of Philosophy
CAOT: Canadian Association of Occupational Therapists
PRMD: Playing-related musculoskeletal disorders
PRMP: Playing-related musculoskeletal problems
ROM: Range of Motion
RAND-12: The 12-question version of the RAND Corporation quality of life measure
SCI: Stress-Coping-Inventory
DASS-21: Depression Anxiety Stress Scales, 21-question version
SRT: Sitting-Rising-Test
KMPAI-R: Kenny Music Performance Anxiety Inventory—revised
MPIIQM: Musculoskeletal Pain Intensity and Interference Questionnaire for Musicians
PA: Physical Activity
OR: Odds Ratio
MCS: Mental Composite Score (of the RAND-12)
PCS: Physical Composite Score (of the RAND-12)
