# Supplementary material for: MusicCohort: Pilot feasibility of a protocol to assess students’ physical and mental health in a Canadian post-secondary school of music
Source: BMC Res Notes. 2021 Dec 4;14:441. doi: 10.1186/s13104-021-05829-9 (PMC8642914; doi:10.1186/s13104-021-05829-9)
Supplement: Supplementary file 1 — Additional file 1. Participant Questionnaire. [file 13104_2021_5829_MOESM1_ESM.pdf]

Participant Questionnaire MusicCohort Study

Inclusion criteria: Over 16 years of age, enrolled in MUSIC 125 (Applied Music)

Exclusion criteria: Regular consumption of pain medication, or medications for mental health diagnosis

General information

Please tick the box or fill in the blanks

1. Code: **(Research Staff Only)**

2. Age: _____

BMI: (Research Staff Only)

3. Gender: F ☐ M ☐ Other ______________

4. Height:__________cm

5. Weight: __________kg *or* _______lb

6. First instrument: ______________________________

7. Second instrument: ______________________________

8. How many years have you played this instrument? First instrument: ______# of years
Second instrument:______ # of years

9. Personal practice duration: First instrument: ________per week
Second instrument: ________per week

10. Typical weekly workload, excluding personal practice: (list per category)

	practice hours per week	performance hours per week
Orchestra or band		
Solo		
Chamber music		
Gigs		
Other		

11. Do you teach music? Yes ☐ No ☐

If yes, how many hours a week do you teach? _________________
How many hours of playing do you do while teaching? ________________

12. Degree program: ________________________

13. Course hours per week (incl. labs): __________________________

Information about everyday life

14. Handedness Writing: right hand ☐ left hand ☐
Throw ball: right hand ☐ left hand ☐

15. Nightly sleep: average of _______hours of sleep per night

16. Please rate your nutrition by circling a number.

0 1 2 3 4 5 6 7 8 9 10

(0=very unhealthy, 10=very healthy)

17. Do you smoke?

- ☐ Never
- ☐ In the past:______ cigarettes per day/ ______years of smoking
- ☐ Yes, ______ cigarettes per day, for _______years

18. Do you drink alcohol?

- ☐ Never
- ☐ Yes, an average of _____glasses per week

19. Do you engage in physical activity?

- ☐ Never
- ☐ Yes, an average of _____hours per week

Which activity/ies? ____

____

20. Which other hobbies do you engage in regularly? ____

____

____
