# Supplementary material for: MusicCohort: Pilot feasibility of a protocol to assess students’ physical and mental health in a Canadian post-secondary school of music
Source: BMC Res Notes. 2021 Dec 4;14:441. doi: 10.1186/s13104-021-05829-9 (PMC8642914; doi:10.1186/s13104-021-05829-9)
Supplement: Supplementary file 2 — Additional file 2: S1. Paper-based assessments. S2. Physical Assessments. S3. Equipment required for assessment protocol. S4. Additional details regarding recruitment and inclusion/exclusion criteria. S5. RAND12 results for total sample and by cohort. S6. Range of motion. S7. Mechanosensitivity, measured by mechanical pressure pain threshold. [file 13104_2021_5829_MOESM2_ESM.docx]

**Additional file 1**

**S1. Paper-based assessments**

The original participant questionnaire was translated into English by a native German speaker who is fluent in both languages. This questionnaire asked about music students’ exposure to musical activities (e.g. how many hours do you practice your first instrument per week; how many years have you played your first instrument) along with other relevant demographic information (e.g. gender; age) and physical activity level, as well as self-reported nutrition. For more information on the participant questionnaire please see Ballenberger et al. [12].

The RAND 12 questionnaire, which is free and does not require a license, was used to address health-related quality of life. A custom scoring algorithm more appropriate for chronic conditions was employed [22]. Test-retest reliability is reported to be excellent (ICC=0.78) and adequate (ICC=0.60) for physical and mental health composites, respectively [51]. The Depression Anxiety Stress Scales (DASS-21) was also administered, which has been translated into multiple languages and is available for use without restrictions [20]. The DASS-21 distinguishes between depression, anxiety, and stress [20], and demonstrates good internal consistency of 0.82-0.97, for both clinical and non-clinical populations [52,53].

Music students answered two additional tools. The Musculoskeletal Pain Intensity and Interference Questionnaire for Musicians (MPIIQM) [23] contains 13 questions and generates sub-scores for pain interference (0-40) and pain intensity (0-50). It is the only validated tool to evaluate playing-related musculoskeletal problems (PRMPs), and the definition used varies slightly from Zaza’s definition of PRMD [1]: the MPIIQM uses the word ‘problems’ rather than ‘disorders’, and employs a definition which includes the statement that PRMPs do not include mild/transient aches and pains. In addition, the MPIIQM has only been validated with professional orchestral musicians. Thus, the first two sections of the questionnaire, which ask questions such as “do you work full-time” and “for how many years have you played professionally”, were removed and replaced with equivalent questions for music students in the participant questionnaire, appended to this paper. The MPIIQM is freely available on the author’s website [54]. There are no cut-off scores established. It has adequate internal consistency and test-retest reliability [23].

Music students also completed the Kenny Music Performance Anxiety Inventory - Revised (KMPAI-R) [21]. This is the only validated tool that measures cognitive, behavioral, and physiological aspects of performance anxiety, and also “underlying psychological vulnerability” [55]. The revised version contains 40 items in eight domains. Participants can score a maximum of 240 points, where a higher score indicates more severe symptoms. It has shown excellent internal consistency (Cronbach's Alpha =0.94), and is freely available on the author’s ResearchGate site [56].

**S2. Physical Assessments**

*Core endurance*

Core strength was assessed using full plank, lateral planks, and the Biering-Sørensen test [57]. Both tests have excellent inter-rater reliability (ICC = 0.80-0.99 and ICC = 0.95, respectively) [58,59]. Tests were first demonstrated, and participants were then asked to hold each position until fatigued or needing to stop for any reason. A 30-second break between tests was given to limit confounding fatigue. Any inappropriate position was verbally corrected once, and further position changes ended the test.

*General mobility*

To assess general mobility/hypermobility, we used the Beighton score and the Sitting-Rising Test (SRT). The Beighton assigns a score of 0 to 9 for four bilateral movements [24]. The SRT assesses general mobility out of 10-points. Participants were asked to sit cross-legged on the floor, and rise with minimal support [25,26]. For any additional support needed, points were deducted. Both tests have good intra- and inter-rater reliability [24,59].

*Mechanosensitivity*

Mechanical pressure pain threshold was assessed using the same nine bilateral testing points as the original protocol [12]. For the first cohort, a mechanical algometer was employed (Pain Diagnostics & Thermography, Italy), which was exchanged for a digital model for subsequent cohorts (FDIX Wagner Instruments, Greenwich, CT, USA). Each point was tested three times, with a 5-second recovery period.

**S3. Equipment required for assessment protocol**

1. Massage table (plinth) – foldable, if assessment is to take place off-site
2. Goniometer
3. Ruler
4. 2 straps for securing participants to the table during Biering-Sorensen test
5. Chair for support of client’s upper body, prior to and after Biering-Sorensen test
6. Stopwatch (or cell phone app)
7. Yoga mat or similar, for comfort during plank and Sitting Rising tests
8. Cervical range of motion (CROM) device
9. Scale for calculation of BMI
10. Device for height measurement (we used a tape measure secure to the wall)
11. Materials for cleaning equipment

**S4. Additional details regarding recruitment and inclusion/exclusion criteria**

Recruitment

Participants were recruited through posters displayed across campus, social media (Twitter, Facebook), campus-wide email, and flyers distributed in-person during a brief presentation in a first-year music class. Participants were informed that they would receive a $5 coffee gift card and one additional gift card for referring other participants. Participants were tested in a university research lab. On testing day, the procedure was explained and participants provided written informed consent. Paper-based and physical assessments were then administered (detailed below). Testing took approximately 70 minutes. All assessments were performed by a trained physio- or occupational therapist, or by occupational therapy students in the second year of a 2-year program. Assessors engaged in a minimum of 2 hours of instruction/training.

The assessment protocol used in this pilot study adhered as closely as possible to the protocol used in the German study [3]. Refinements to testing were made through continuous communication with the investigators in Germany. This paper reports findings of the final protocol; required changes are addressed in the discussion.

Inclusion criteria

1. first-semester, first year student majoring in music: First-semester refers to the stage of their program (the students were in their first semester of study at university). Full-time, first year student majoring in music refers to students in Bachelor of Music programs. Many music programs in Canada only accept full-time students in Bachelor of Music programs. This criterion was intended to exclude students who might take a few courses, or do a Bachelor of Arts degree with a minor in music. In many cases, non-music majors and students who are not full-time are not eligible to enroll in performance courses (courses where a student receives a grade for playing an instrument or singing). Taking a performance course for credit was also an exclusion criterion for students who were in the control group, in order to make a clear division between the two groups.

Exclusion criteria

1. *Varsity athletes*: Varsity (inter-university competitive) sports are prominent in North America. Some students are admitted to post-secondary institutions based on their ability to compete in varsity sports. Varsity sports demand significant time committment, and athletes are typically in excellent physical condition. Since many post-secondary music programs in Canada require that students participate in ensembles, which may or may not be counted as courses, it is rare that music students have the time to engage in varsity sports. Thus, varsity athletes are more likely to be in the non-music control group. Their presence might therefore skew the data on physical aspects such as core strength, mechanosensitivity, and in some cases, range of motion. Therefore, we excluded varsity athletes from our study.
2. *Diagnosed neurological, orthopaedic, or psychological condition, infections or systemic disease; regular medication for pain or mental illness*: These exclusions were included in the German parent study. Their purpose was to ensure that the assessment did not aggravate any medically diagnosed conditions, since the assessment was not intended to treat these pre-existing conditions. In addition, we excluded students who regularly consumed medication for pain or for mental illness. This was to reduce outliers in the data. These were assesed by self-report, by asking participants whether they had a diagnosis of any of these conditions, or were taking any medication regularly for pain or mental illness. If students indicated that they took pain medication occasionally for headaches, for example, we would not have excluded these; whereas daily consumption would exclude them from participating. More detail can be found in the participant questionnaire, which gives wording for this exclusion; and the two publications arising from the parent study [12,34].

**S5. RAND12 results for total sample and by cohort**

| **RAND12** | **Overall (N=69)** | **Musicians (N=19)** | **Non-Musicians (N=50)** | **Statistics** |
| --- | --- | --- | --- | --- |
| **Physical functioning** | 55.95 (8.50) | 55.95 (8.50) | 55.95 (8.50) | p= 0.560,  r=-0.560,  Z=-0.575 |
| **Role physical** | 57.17 (9.51) | 52.42 (9.51) | 57.17 (5.94) | p= 0.190,  r=-0.159,  Z=-0.190 |
| **Bodily pain** | 57.27 (9.41) | 47.86 (9.41) | 57.27 (0.00) | **p=** **0.004***,  r=-0.348,  Z=-2.887 |
| **General health** | 55.49 (10.38) | 45.11 (10.38) | 55.49 (10.38) | p= 0.053,  r=-0.231,  Z=-1.918 |
| **Vitality (energy)** | 44.91 (10.38) | 44.91 (20.77) | 44.91 (10.38) | p= 0.170**,**  r=-0.167,  Z=-1.391 |
| **Social functioning** | 56.17 (10.12) | 56.17 (10.12) | 56.17 (10.12) | p= 0.459,  r=-0.092,  Z=-0.770 |
| **Role emotional** | 44.98 (17.34) | 39.20 (17.34) | 44.98 (13.00) | p= 0.175,  r=-0.164,  Z=-1.365 |
| **Mental health composite score** | 48.99 (10.76) | 44.68 (14.67) | 54.74 (5.97) | p= 0.169,  r=-0.171,  Z=-1.424 |
| **Physical health composite score** | 53.90 (6.29) | 51.60 (5.91) | 49.50 (10.59) | p= 0.055  r=-0.231  Z=-1.921 |

*Values present median and Interquartile Range for RAND12 scores, test statistics for Mann-Whitney-U-test,* α=0.005, **Bold *** *indicates* α *<0.005.*

**S6. Range of motion**

| **Movement** | **All (N=69)*** | **Musician (N=19)*** | **Control (N=50)*** | **Statistics** |
| --- | --- | --- | --- | --- |
| Mean CROM | 59.17 (10.08) | 59.83 (11.00) | 58.97 (10.33) | p=0.648,  r=-0.077,  Z=-0.643 |
| Mean ROM | 74.44 (8.25) | 74.31 (7.31) | 74.69 (8.55) | p=0.486,  r=-0.085,  Z=-0.705 |

**Values represent median and interquartile range of range of motion in degrees, test statistics for Mann-Whitney-U-test,* **Bold *** *indicates* α=0.005, *r = effect size.*

**S7. Mechanosensitivity, measured by mechanical pressure pain threshold**

| **Test point** | **All (N=69)*** | **Musician (N=19)*** | **Control (N=50)*** | **Statistics** |
| --- | --- | --- | --- | --- |
| R Trapezius | 45.30 (26.69) | 41.84 (29.00) | 47.30 (28.67) | p=0.200,  r=-0.155,  Z=-1.290 |
| L Trapezius | 42.17 (33.40) | 37.10 (17.49) | 46.48 (35.41) | **p=0.050***,  r=-0.236,  Z=-1.961 |
| R Supraspinatus | 47.33 (34.47) | 40.54 (22.17) | 49.39 (40.83) | p=0.227,  r=-0.147,  Z=-1.216 |
| L Supraspinatus | 44.70 (28.89) | 35.00 (26.21) | 51.09 (32.41) | **p=0.008***,  r=-0.315,  Z=-2.619 |
| R Wrist Extensor | 39.40 (17.88) | 30.40 (15.53) | 40.59 (17.14) | **p=0.023***,  r=-0.272,  Z=-2.257 |
| L Wrist Extensor | 31.20 (19.36) | 29.00 (11.20) | 35.50 (22.01) | p=0.184,  r=-0.161,  Z=-1.337 |
| R Wrist Flexor | 51.50 (28.86) | 41.68 (27.40 | 56.75 (26.51) | **p=0.021***,  r=-2.297,  Z=-0.277 |
| L Wrist Flexor | 40.83 (23.08) | 33.70 (19.94) | 42.77 (22.06) | **p=0.030***,  r=-0.260,  Z=-2.163 |
| R Temporalis | 23.17 (12.92) | 18.96 (9.02) | 25.05 (12.02) | **p=0.009***,  r=-0.310,  Z=-2.573 |
| L Temporalis | 21.00 (10.00) | 19.29 (7.00) | 21.58 (11.00) | p=0.169,  r=-0.167,  Z=-1.384 |
| Mean algometer | 37.43 (15.28) | 30.53 (11.20) | 38.76 (16.29) | **p=0.029***,  r=-0.265,  Z=-2.190 |

**Values represent median and interquartile range in Newtons, statistics for Mann-Whitney-U-test,* α=0.005, **Bold *** *indicates* α *<0.005, r = effect size.*
